# Phytoplankton Toxins and Their Potential Therapeutic Applications: A Journey toward the Quest for Potent Pharmaceuticals

**DOI:** 10.3390/md20040271

**Published:** 2022-04-18

**Authors:** Biswajita Pradhan, Jang-Seu Ki

**Affiliations:** Department of Biotechnology, Sangmyung University, Seoul 03016, Korea; pradhan.biswajita2014@gmail.com

**Keywords:** phytoplankton, toxins, therapeutic, pharmaceuticals

## Abstract

Phytoplankton are prominent organisms that contain numerous bioactive substances and secondary metabolites, including toxins, which can be valuable to pharmaceutical, nutraceutical, and biotechnological industries. Studies on toxins produced by phytoplankton such as cyanobacteria, diatoms, and dinoflagellates have become more prevalent in recent years and have sparked much interest in this field of research. Because of their richness and complexity, they have great potential as medicinal remedies and biological exploratory probes. Unfortunately, such toxins are still at the preclinical and clinical stages of development. Phytoplankton toxins are harmful to other organisms and are hazardous to animals and human health. However, they may be effective as therapeutic pharmacological agents for numerous disorders, including dyslipidemia, obesity, cancer, diabetes, and hypertension. In this review, we have focused on the properties of different toxins produced by phytoplankton, as well as their beneficial effects and potential biomedical applications. The anticancer properties exhibited by phytoplankton toxins are mainly attributed to their apoptotic effects. As a result, phytoplankton toxins are a promising strategy for avoiding postponement or cancer treatment. Moreover, they also displayed promising applications in other ailments and diseases such as Alzheimer’s disease, diabetes, AIDS, fungal, bacterial, schizophrenia, inflammation, allergy, osteoporosis, asthma, and pain. Preclinical and clinical applications of phytoplankton toxins, as well as future directions of their enhanced nano-formulations for improved clinical efficacy, have also been reviewed.

## 1. Introduction

Epidemiological studies have found that modern diets, alcohol, and antibiotic consumption increase the risk of oxidative damage, which lead to diseases such as inflammatory diseases, cancer, aging, coronary heart disease, cardiovascular disease, and other ROS-related diseases [1,2]. In addition, several new diseases related to microbial pathogens are occurring [3]. In recent times, such dangerous diseases are on the rise as a result of rapid urbanization and lifestyle changes. Chemotherapy and other medications used in cancer treatment have side effects, especially in terms of drug tolerance. Moreover, microorganisms are becoming resistant to drugs due to conventional drug treatments [3]. In this regard, searching for novel drugs from natural sources can resolve this issue [4].

Efforts to extract pharmaceuticals from natural sources commenced in the late 1960s. To date, approximately 2500 novel metabolites have been discovered in a range of species. These studies showed that marine and freshwater environments are a great source of novel compounds that do not originate from terrestrial sources. More than 10,000 chemicals have been identified from both marine and freshwater species, and over 300 patents on bioactive natural products have been allotted [5].

Phytoplankton are photosynthetic organisms found in large numbers in aquatic environments, worldwide. This diverse collection of phytoplankton accounts for about half of worldwide CO_2_ fixation and is the foundation of the aquatic food chain. Phytoplankton play an important role as primary producers not only in freshwater ecosystems, but also in marine ecosystems [6,7,8,9,10].

Phytoplankton are potentially the most novel source of bioactive secondary metabolites, including toxins. They display antioxidant, anticancer, antibacterial, antifungal, antiviral, antidiabetic, anti-inflammatory, and other activities that can be employed in drug development and treatment [2,11,12,13]. Phytoplankton are responsible for the production of harmful toxins [14,15,16]. Although phytoplankton toxins can be hazardous to aquatic ecosystems and human health, some aquatic organisms are not affected by the toxins and may even contribute to several biomedical applications [17]. Not only cancer, but also diabetes, inflammation, and ROS-related diseases are among the most common health concerns in the United States and other countries, and no satisfactory treatment strategies are currently available [1,18]. The currently used synthetic medications and other therapies have a variety of adverse effects that are detrimental to health. Consequently, in recent times, alternative remedies have been sought, and natural products are being investigated [3,11,19]. Plants are the most important source of natural molecules, which have taken a lead in pharmaceutically created moieties in this “synthetic age”. Natural chemicals derived from medicinal plants are becoming increasingly essential in the treatment of cancer and other diseases due to the various harmful side effects of current cancer treatments. More than half of all pharmaceuticals in clinical use around the world are natural substances and derivatives, and over 60% of cancer treatments approved are of natural origin [20,21,22,23]. However, marine phytoplankton may be a source of novel secondary metabolites, such as toxins, which may have potential biological applications [12]. Pioneering studies on drug discovery from marine phytoplankton have been conducted in recent decades.

Therefore, this review is based on several phytoplankton toxins and their potential biological applications. In addition, it also provides new research for the discovery of new drugs for life-threatening diseases. Drug synergism, in the present era of discovery of new drugs, focuses on preclinical and clinical applications, pharmacodynamics, pharmacokinetics, and enhanced drug delivery technologies to produce next-generation tailored treatments for disease prevention. The use of toxins will open up new frontiers in studies related to therapy for various diseases once we understand the possible molecular key players involved. Finally, such toxins could serve as therapeutic drugs in the near future.

## 2. Phytoplankton: The Most Ingenious Source of Toxins

Phytoplankton are among the most important components of aquatic ecosystems [6,24]. They not only serve as a foundation for all aquatic food chains, but also provide a valuable service to humans and other living creatures by producing a large amount of oxygen after absorbing carbon dioxide from the environment [6]. Phytoplankton are buoyant and float on the upper surface of water bodies. However, they are similar to terrestrial plants in that they both have chlorophyll and require sunlight to survive and thrive. They also require inorganic nutrients, including phosphates, sulfur, and nitrate, which are converted into lipids, carbohydrates, and proteins [6].

**Figure 1:**
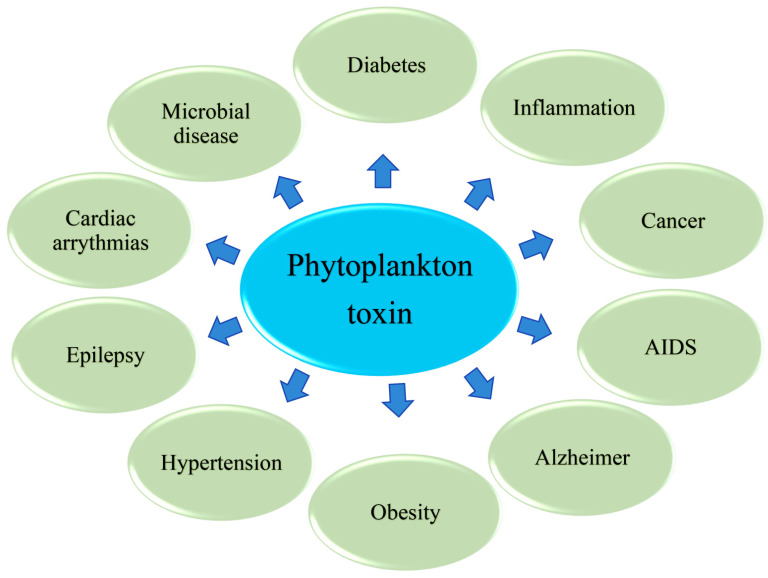
Phytoplankton toxin modulates different diseases in human.

Many phytoplankton species produce compounds that are poisonous to humans, which is why they are called “toxic microalgae”. Phytoplankton toxins have a variety of chemical structures, ranging from relatively simple alkaloids and amino acids to polyketides. Polyketides belong to a family of extremely diverse compounds in terms of structure and potential biological properties. However, the evolution and functional therapeutic relevance of these secondary metabolites remains unknown. Therefore, this review focuses on the effects of toxins and their crucial roles in disease prevention. Phytoplankton toxins that modulate different diseases in humans are displayed in Figure 1.

## 3. Phytoplankton Toxins Kills to Heal: A Cross Talk

For decades, the creation of toxins by phytoplankton has piqued the interest of the scientific community. Thousands of articles have been published in the field of algal production of toxins. Meanwhile, the ecological role of phytoplankton poisons such as hepatotoxins and neurotoxins produced by cyanobacteria, diatoms, and dinoflagellates is still up for debate in relation to their pharmacological applications. Numerous hypotheses have been proposed that suggest a modest advantage or physiological function for the production of toxins [25]. Phytoplankton toxins can have a great ability to prevent disease in mammals and humans. Several studies have been published, and much has been discussed about the deleterious consequences of phytoplankton toxins on human health. However, many questions still remain unanswered about the true impact of these substances on humans [26,27,28].

Microcystins from cyanobacteria have been shown to cause acute hepatotoxicity by inhibiting protein phosphatases (PP1 and PP2A) and oxidative stress, as well as by acting as tumor promoters by deregulating mitogen-activated protein kinases and activating protooncogenes [29,30,31,32]. In addition, microcystins have been proposed to be genotoxic agents and, as a result, are tumor initiators in humans [33]. However, no evidence of a link between microcystins and the development of cancer in humans has been revealed yet. As a result, it is currently unknown how to assess this link in order to control microcystin risks [34]. Furthermore, epidemiological studies showed associations in organoid, tissue, and cell culture. In addition, some studies on animals showed some associations. Hence, health guidelines are available worldwide. Key research areas for phytoplankton toxins include pharmacology (17%), chemistry (18%), ecology (19%), and toxicology (24%) [35]. More than 90% of global research on phytoplankton toxins demonstrates that the existence and properties of such toxins are not properly analyzed and are most likely underestimated [35]. However, there is some evidence to suggest that phytoplankton toxins can be used in pharmacology in the near future [36].

**Figure 2:**
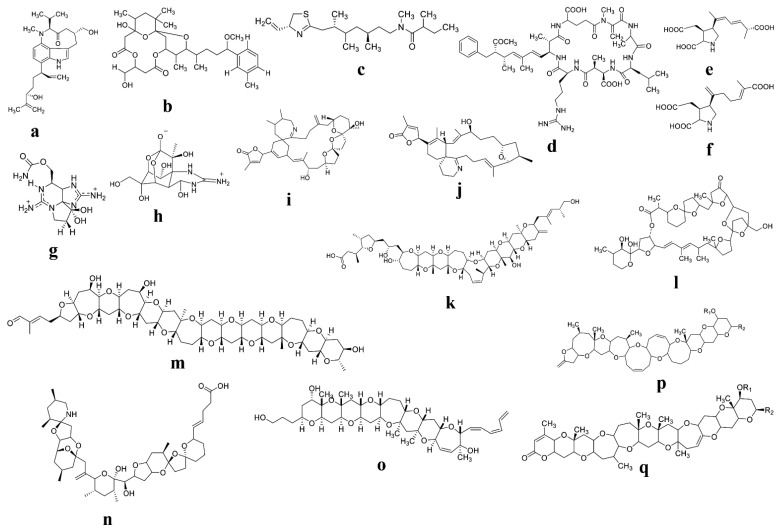
Molecular structure of various phytoplankton-derived toxins with potential therapeutic effects: (**a**) Lyngbyatoxin; (**b**) Oscillatoxins; (**c**) Kalkitoxin; (**d**) Microcystin; (**e**) Domoic acid; (**f**) Iso-domoic acid; (**g**) Saxitoxin (STX); (**h**) Tetrodotoxin (TTX); (**i**) gymnodimines A (GYMA); (**j**) 13-desmethyl spirolide C; (**k**) Ciguatoxin; (**l**) Pectenotoxin; (**m**) gymnocin-A (GYMA; (**n**) Azaspiracid-1 (AZA1); (**o**) Gambierol; (**p**) Brevetoxin type-A; (**q**) Brevetoxin type-B.

**Figure 3:**
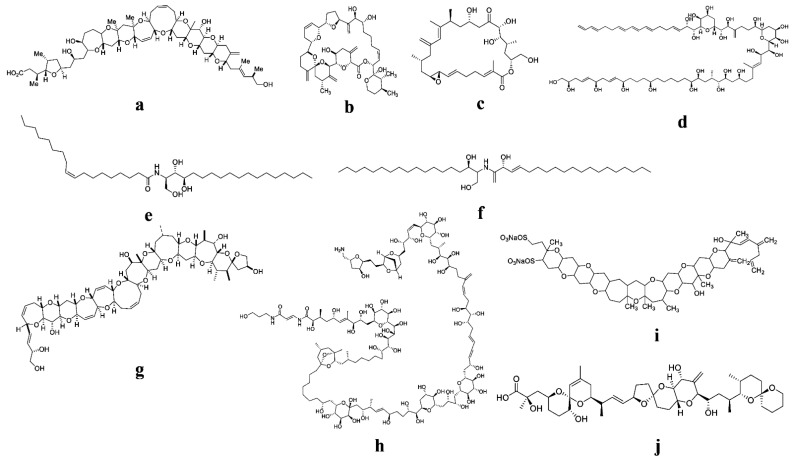
Molecular structure of various phytoplankton-derived bioactive compounds and toxins with potential therapeutic effects: (**a**) Gambieric acid; (**b**) Goniodomin-A; (**c**) Amphidinolide-H; (**d**) Amphidinol-3 (AM3); (**e**) Ceramide; (**f**) Symbioramide; (**g**) Ciguatoxin; (**h**) Palytoxin; (**i**) Yessotoxin; (**j**) Okadaic acid.

The search for biologically active secondary metabolites with therapeutic potential is not new. Since ancient times, nature has been recognized as a valuable source of possible medicines. Toxins such as digoxin, paclitaxel, morphine, atropine, and colchicine are among the first biologically active chemicals to be identified, and they are still used in medical practice and healthcare systems today [37]. Many other life-saving medications, such as antibiotics, immunosuppressants, and anticancer drugs, have been extracted from natural sources. Even today, natural ingredients are the source of the bulk of novel chemical entities offered as medications in the market [38]. This explains why these compounds continue to play such an important role in the development of new front-line medications. Hundreds of phytoplankton compounds have been found recently, many of which have antiviral, antibacterial, anticancer, and other properties [39]. Despite their powerful biological actions, only few molecules have entered clinical trials, and none of them are phytoplankton-derived. In this regard, toxins that cause paralytic poisoning and other toxins that have been established as highly toxic compounds may be suitable candidates for pharmaceutical purposes. The use of phytoplankton toxins may ultimately develop a new drug for combating human diseases in the near future. In some cases, the use of toxins for medicinal purposes has already been proven [40]. Nevertheless, it is now one of the most versatile medications used to treat human disorders in the fields of ophthalmology, neurology, and dermatology [40,41]. To the best of our knowledge, a few marine algae-derived bioactive compounds, such as phlorotannins, polysaccharides, fucoidans, alginic acid, tripeptides, pyropheophytin, and oxylipin, have been shown to reduce the risk of cancer, diabetes, and inflammatory diseases. In this regard, phytoplankton toxins can be used as therapeutic agents and have been established as potent pharmacophores against stress-associated diseases in humans. Phytoplankton toxins and their different potential therapeutic applications are displayed in Table 1. Various biological activities and chemical structures of phytoplankton toxins are shown in Figure 2 and Figure 3**.**

## 4. Toxins Produced by Cyanobacteria and Their Potential Biomedical Applications

**Figure 4:**
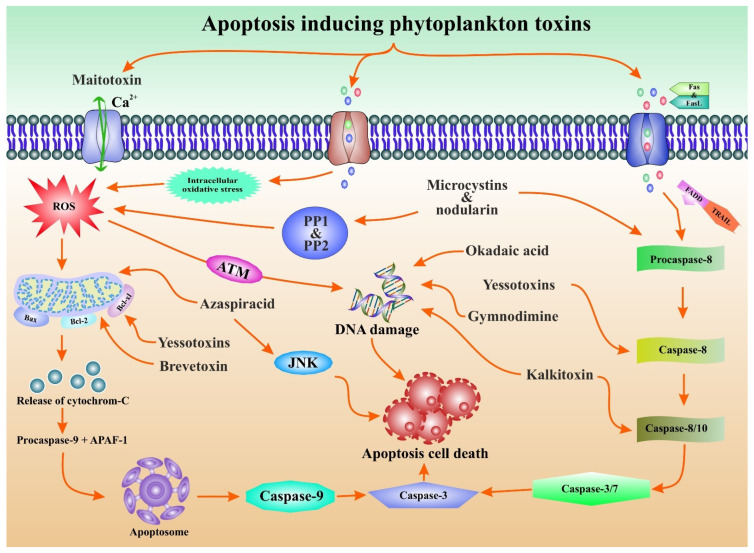
Apoptosis modulation by phytoplankton toxin in cancer prevention. Phytoplankton toxins such as azaspiracid, yessotoxins, and brevetoxins cause intracellular oxidative stress, which leads to mitochondrial dysfunction and downregulates the expression of anti-apoptotic proteins Bcl-xl and Bcl-2. Similarly, they enhance Bax expression to aid apoptosis via release of cytochrome-C, which triggers the formation of apoptosomes, leading to caspase 9 and 3 being induced and displaying apoptotic cell death. Microcystins and nodularin trigger the induction of caspase and display caspase-dependent apoptotic cell death. Moreover, microcystins and nodularin inactivate PP1 and PP2, which leads to excessive ROS. Excessive ROS enters into the ATM signaling pathways, which leads to DNA damage and displays apoptotic cell death. In addition, yessotoxins, gymnodimine, kalkitoxin, and okadaic acid trigger DNA damage, leading to apoptotic cell death. Kalkitoxin also induced the activation of caspase 8/10 and caspase 3/7, displaying caspase-dependent apoptotic cell death. Moreover, Azaspiracid entered into the JNK pathway and displayed apoptotic cell death.

Cyanobacteria offer a variety of chemical compounds that have received much interest, especially in medical chemistry and pharmacology [89]. They produce a wide array of structurally diverse and bioactive compounds with anticancer, antibacterial, antifungal, antituberculosis, immunosuppressive, antioxidant, and anti-inflammatory activities [90,91,92]. Conversely, many cyanobacterial toxins have anticancer potential in various cell lines, providing hopeful results for future study into human glandular cancer management [93]. Phytoplankton toxins displayed potent anticancer activity via apoptosis modulation for cancer treatment and prevention (Figure 4).

Natural products’ capacity to stop cancer cell lines from growing could lead to the development of effective anticancer drugs [23]. In this regard, there are several cyanobacteria that belong to potential groups of various taxa of marine organisms of pharmaceutical value, for example, *Leptolyngbya*, *Lyngbya*, *Microcystis*, *Oscillatoria*, *Calothrix*, *Symploca*, *Dichothrix*, *Schizothrix*, *Geitlerinema*, *Aphanothece*, *Synechocystis*, and *Blennothrix* [94]. Chemical compounds having anti-proliferative, anti-cancer, and anti-tumor activities via apoptotic death or influencing cell signaling via induction of protein kinase-C (PKC) were found in abundance in marine *Lyngbya majuscula* [95,96]. Nair and Bhimba investigated the anticancer activities of the cyanobacteria *Oscillatoria* spp., and the results showed that *Oscillatoria boryana* has anticancer activity against human breast cancer cell lines [97]. Targeting HIF-1 (hypoxia-inducible factor-1) and processes downstream of mitochondrial respiration is an evolving topic in the pharmacology of cytotoxins from marine cyanobacteria [98]. Oftedal et al. discovered that extracts from *Anabaena* sp. M44, M30, and M27 rapidly trigger apoptosis by comparing the higher-than-therapeutic concentration of daunorubicin in cells from AML (acute myeloid leukemia) in humans [99].

Cyanotoxins are a rich source of naturally occurring cytotoxic compounds that can target tumors by inducing the expression of specialized uptake transporters. Combinatorial engineering, due to its structure, can be used to advance the therapeutic index and address organ-specific toxicity concerns [100]. Furthermore, oscillatoxin and its analogs, such as oscillatoxin E and 30-methyloscillatoxin D from the cyanobacterium *Lyngbya* sp., inhibited Kv1.5 expression in CHO cells with IC_50_ values of 0.79 ± 0.032 and 1.47 ± 0.138 µM, respectively. These findings should be useful to researchers looking for new ways to treat atrial tachyarrhythmias [101]. Kalkitoxin from *Lyngbya majuscula* displayed cytotoxicity against HCT-116 colon cell lines [102]. Furthermore, it reduces hypoxia-induced initiation of HIF-1 in T47D breast tumor cells with an IC_50_ value of 5.6 nM [102]. Moreover, because kalkitoxin interacts with voltage-sensitive sodium channels, it inhibits calcium influx inhibition in primary rat cerebellar granule neuron cultures [103]. OATP (organic anion transporting polypeptides)–microcystin interaction as a potential anti-cancer regimen is risky since OATPs are also expressed in healthy cells; treatment must be targeted locally. Aside from the liver, microcystins can also have detrimental effects on other organs such as the heart, kidney, and brain. Microcystins and nodularin are stable and have the potential to cause cellular damage after uptake via organic anion transporting polypeptides (OATPs) by causing intracellular inhibition of the catalytic subunit of PP1 (protein phosphatase 1) and PP2, glutathione depletion, and the generation of ROS (reactive oxygen species) [89,104]. Because certain OATPs are overexpressed in tumors compared to normal tissues, microcystins could be interesting targets for anticancer drug development [89]. Cancer cells have a high level of intrinsic oxidative stress, making them vulnerable to exogenous ROS assaults. As a result, analogues of microcystin can kill cancer cells that express OATP while inflicting little harm on healthy cells [105]. Microcystins and nodularin decrease PP1 and PP2A activity and induce apoptosis in human embryonic kidney HEK 293, Swiss 3T3 mouse embryo fibroblast, breast carcinoma cell line MCF-7, and rat promyelocytic IPC-81 leukemia cells via cell shrinkage, membrane blebbing, and organelle relocation [106,107,108]. More research is required for the exploration of cyanotoxin in the field of pharmacology to establish a new pharmacophore against deleterious diseases such as cancer in humans.

## 5. Diatom’s Toxins: The Legendary Furthest Effective Biological Properties

Diatoms are the most common photosynthetic organisms in the world’s oceans and are critical for energy transfer through marine food chains. However, multiple studies have revealed that they produce hazardous secondary metabolites [109]. Toxigenic diatoms cause poisoning in both people and animals on a regular basis [110]. Not only the genus *Pseudo-nitzschia* but also *Amphora* have been identified as domoic acid (DA) producers, which is a nonprotein amino acid that is water soluble, crystalline, and has a molecular weight of 311 Da [111,112]. Recently, Antarctic diatom species have been identified as DA producers [113]. Less toxic isomers of DA have been discovered, such as isodomoic acid A and isodomoic acid B from *Nitzschia navis-varingica* and isodomoic acid C from *Pseudo-nitzschia australis* [114,115,116]. Humans and nonhuman primates showed no hazardous symptoms at low doses of DA (0.20e0.75 ppm), but clinical effects were seen at 1.0 ppm, and the tolerated daily intake (TDI) of DA for humans was calculated to be 0.075 ppm. The TDIs for razor clams and crabs were 19.4 and 31.5 ppm, respectively [109]. Conversely, although DA has poisoned humans, fish-eating birds, and marine mammals, the DA has several disease preventive actions against several deleterious diseases such as cancer [117]. DA was responsible for the natural medicine’s curative effectiveness in killing parasitic worms [111], and it displayed proliferative effects on cancer cell lines such as K562 and EA.hy 927 in vitro [118]. Although little research has been conducted on the disease-preventive effects of DA, more research is needed in order to develop a new drug molecule to combat harmful diseases in humans.

## 6. Dinoflagellate Biologically Active Toxins and Their Potential Biomedical Applications

Dinoflagellates are unicellular and planktonic and are a promising source of biologically active toxins that have an impact on the safety of seafood and human health. Due to HABs, dinoflagellates have been identified as potent natural physiologically active toxin makers in marine environments [17]. The dinoflagellate toxin not only harms the marine environment, but it is also detrimental to economic activities (such as aquaculture, fisheries, and tourism) [119]. Despite the disadvantages listed above, dinoflagellate toxins are valuable and interesting molecules due to their unusual structure and wider functioning. The potential of dinoflagellate-derived toxins as attractive pharmacological effectors and/or biological investigative probes has been revealed in several biological studies [120,121,122]. Toxins from dinoflagellates and their different potential therapeutic applications are displayed in Supplementary Table S1.

### 6.1. Dinoflagellate Toxins: The Most Prevailing Source of Toxins with Biological Properties

Dinoflagellate toxins and their analogs are employed in medical research in order to better understand their mechanisms of action and assess their therapeutic potential. These toxic marine dinoflagellates, such as the genera *Alexandrium* (*A. minutum*, *A. catenella*, *A. tamarense*), *Pyrodinium* (*P. bahamense*), and *Gymnodinium* (*G. catenatum*), produce saxitoxin (STX) and its naturally occurring equivalents (neosaxitoxin and gonyautoxins). Other sources of STX-group toxins, such as cyanobacteria, have been identified, including the genera *Anabaena*, *Lyngbya*, *Planktothrix*, *Aphanizomenon*, and *Cylindrospermopsis* [123]. Saxitoxin (STX) is a group of toxins (also known as paralytic shellfish poisons, or PSPs) produced by cyanobacteria in freshwater and dinoflagellates in marine water. STX is grouped into non-sulfated (neoSTX), mono-sulfated (GTX1-6), di-sulfated (C1-4), decarbamylated (dcSTX, dcneoSTX, dcGTXs1-4), and deoxy-decarbamoylated (doSTX, doGTXs1-3) with varying toxicities. These toxins primarily block the sodium channels in the nerve and muscle cells, resulting in paralysis. They also act as potential therapeutics, such as anesthetic agents. They may minimize or even block pain sensations, muscle spasms, muscle relaxation, and wrinkle reduction. STXs possess promising antifungal, antibacterial, antialgal, and antiprotozoal activity in vitro [88]. *Amphydinium* dinoflagellates produce some important analogues of STXs, such as gonyautoxins (GTXs), which have a similar mode of action. GTXs have been shown as promising and safe therapeutic agents for acute or chronic anal fissures, and they are also used as a pain reliever [73]. Moreover, chronic tension-type headaches have also been treated with GTX2 and GTX3 [74]. The gonyautoxins are the paralytic toxins produced by the dinoflagellate *Amphidinium* that have been employed in clinical practice for anal sphincter infiltrations [73]. The antifungal polyether macrolide goniodomin-A, generated by the dinoflagellate *Goniodoma pseudogoniaulax*, has been demonstrated to prevent angiogenesis by decreasing endothelial cell migration and tube formation caused by basic fibroblast growth factor (bFGF) [124]. Goniodomin-A has active effects in vivo as well [124]. Gymnocin-A, derived from the red tide dinoflagellate *Gymnodinium mikimotoi*, is cytotoxic to P388 murine leukemia cells [49].

Tetrodotoxin (TTX) is produced primarily by bacteria and is recently associated with marine dinoflagellate toxin, which was discovered in pufferfish and is linked to saxitoxins. The toxin is produced by *Alexandrium tamarense* and is a long-acting topical anesthetic regarded as safe and effective [125]. TTX is a highly unique chemical structure and a strong neurotoxin that is of particular interest due to its similarities in effects to saxitoxins (and equivalents). TTX and STX are structurally distinct but with a similar mode of action; both block conductance in voltage-gated sodium channels, resulting in inhibition of neuromuscular signal transmission [126]. TTX poisoning is generally associated with contaminated puffer fish, although such poisonings have also been reported with consumption of shellfish. TTX has also been detected in various vertebrates and invertebrates (e.g., worms, starfish, frogs, newts, octopus, slugs, etc.). TTX has been demonstrated to reduce narcotic hunger in laboratory animals and is being employed in drug addiction [126]. TTX is effective for the treatment of heroin addiction. Moreover, clinical trials resulted in finding that TTX (5 and 10 µg) is also beneficial in relieving opiate withdrawal symptoms with minor side effects [122].

Okadaic acid (OA) and its derivatives, such as dinophysistoxins (DTX)-1, 2, and 3, are lipophilic polyethers of marine biologically active toxins found in a variety of fish and shellfish species. When consumed by humans, they can cause gastrointestinal symptoms (known as diarrheic shellfish poisoning, DSP). These were first isolated from benthic dinoflagellates. OAs are known phosphatase inhibitors, particularly PP1 and PP2, which are similar to microcystins, and are primarily produced by *Prorocentrum* dinoflagellates (*P. lima*, *P. cuncavum*, *P. belezeanum,* and *P. mascul*) and Dinophysis (*D. acuminate*, *D. acuta*, and *D. fortii*) [124,125]. They are a marine biologically active toxins that have been connected to many health problems, such as causing diarrhetic shellfish poisoning, and have proven useful in several cellular processes, making them important in medical study [127]. OA’s value in medical/pharmacological research has been established in numerous studies, both in vitro and in vivo [43]. Numerous in vitro and in vivo studies have shown that OAs have other effects on cellular metabolism, regulation, and control [43]. Okadaic acids are especially useful for studying cellular processes that are regulated by phosphorylation.

In numerous cell types, including blood cells, intestinal cells, hepatic cells, lung cells, and brain cells, OA has displayed cytotoxic effects via apoptosis and inhibition of cell growth. It has cytotoxic effects on embryonic development, the immune system, and the neurological system [43]. Okadaic acid, which inhibits protein phosphatase 2A, is being used in research to clarify the processes by which conjugated linoleic acids may function as anti-tumor mediators in breast cancer cells [39]. Because of its tumor-promoting and cytotoxic properties, okadaic acid is a model potent neurotoxin for studying the therapeutic effects of typical antipsychotic medications in the treatment of cognitive impairment and neuropathological alterations in schizophrenia and other neurodegenerative diseases [72]. Because of its ability to inhibit serine/threonine phosphatases and the protein PP2A, OA has become a promising tool in the study of Alzheimer’s disease (AD) and other neurodegenerative illnesses linked to memory loss. Reduced PP1 and PP2 activity leads to hyperphosphorylation of tau protein, which is a major marker in AD [68]. OA has also been used as a biotoxin model in studies on diabetes, cancer, and AIDS to reveal numerous pathways related to these diseases [76]. Furthermore, OA appears to have immunomodulatory effect potential since it causes T-cell receptor expression to be downregulated, affecting T-cell function in immune responsiveness and, as a result, immunological response [128]. It can also trigger an inflammatory response in HL-60 human cells by significantly increasing interleukin 8 (IL-8) levels [44]. In addition to being a potent tumor promoter, OA has been shown to increase the activity of HIF-1, a protein closely linked to vascular endothelial growth factor in human endothelial cells [45]. Finally, OA from *Prorocentrum* sp. has been demonstrated to have fungicidal properties, specifically the capacity to suppress *Candida albicans* growth [79].

Amphidinolides and colopsinols are two families of macrolides synthesized by marine dinoflagellates of the genus Amphidinium that exhibit powerful anticancer effects via inhibition of cancer cell lines [129]. Biological research has been hampered by the extremely limited availability of these chemicals [46]. In vitro, amphidinolides showed high cytotoxicity against murine lymphoma L1210 and human epidermoid carcinoma KB cells [46]. The human colon tumor cell line HCT 116 and its drug resistant variation, HCT 116/VM 46, have shown high cytotoxicity in response to a similar chemical called caribenolide I [47]. In addition, it is also efficacious in vivo against the mouse tumor P388 [48].

Yessotoxins (YTXs) are sulphated polyethers, a class of marine toxins derived from *Protoceratium reticulatum*, *Lingulodinium polyedra*, and *Gonyaulax spinifera* [117,118]. As a result of this property, such a toxin is regarded as one of the most polar among the otherwise lipophilic toxins [120,130]. YTXs impair the E-cadherin–catenin system selectively in epithelial cells, thus jeopardizing Ecadherin’s tumor-suppressive effects [50]. In the supernatant of the cultivated dinoflagellate Protoceratium cf. reticulatum, Yessotoxins have displayed significant cytotoxic effects [120]. Protoceratins I, II, III, and IV are the four equally active glycoside polyether principles found in the extract. These compounds displayed cytotoxicity selectively against human cancer cell lines with mean IC_50_ values of less than 0.0005 M [53].

In a number of cellular systems, such as tumor cells, YTX and its analogues are particularly interesting tools for studying biological and pharmacological mechanisms with multiple biological apoptotic pathways [131]. YTX also caused non-apoptotic cell death in primary cortical neurons, BC3H1 myoblast cells, and glioma cells [51]. Moreover, it also acts as a potent phosphodiesterase (PDE) activator [132]. PDEs are important regulators of signal transmission, which is mediated by substances such as cyclic adenosine monophosphate (cAMP) and modulates caspase protein inactivation via permeability transition through mitochondria and alteration of the cytoskeleton via selective disruption of F-actin microfilaments [133,134]. It has recently been found to cause mitotic catastrophe and genetic modifications, which may be useful for cancer progress management [52]. Additionally, it also inhibits the growth of melanoma tumor cells in mouse cells in vivo with minimal damage [52]. YTX appears to impair immune function by reducing phagocytic activity in the J774 cell line and increasing cytokine expression in J774 phagocyte mammalian cells [133]. Furthermore, it appears to control the immunological impact on T-lymphocyte EL-4 cells via reversible T-cell receptor complex downregulation [128]. YTX and its analogues could be used to treat Alzheimer’s disease by lowering the levels of t- and β-amyloid, two insoluble formations found in the brain that are accountable for the illness’s onset [69]. Furthermore, YTX may aid in the prevention and treatment of lipid and glucose metabolism-related disorders in glioma cells as well as pancreatic and liver transcriptional abnormalities [135]. YTX may also have a minor role as an anti-asthmatic and anti-allergenic drug [83].

*Dinophysis* species such as *D. tripus*, *D. acuta*, *D. fortii*, *D. caudate*, *D. acuminate*, *D. norvegica*, and *D. rotundata* are reported as producers of pectenotoxins (PCTs) that have anti-cancer effects [136]. Pectenotoxins (PTXs) and their 20 analogues that have been isolated from *Dinophysis* species are strongly cytotoxic against various human cancer cell lines [137]. For example, PTX-2 has been shown to have anticancer action in human lung, colon, and breast cancer cells [54]. Actin inhibitor pectenotoxin-2 (PTX2) has been proposed as a potential chemotherapeutic treatment for p53-deficient malignancies [138].

Ciguatoxin (CTX) is a fat-soluble toxin generated by specific benthic *Gambierdiscus toxicus* and some species of *Gambierdiscus* such as *G. belizeanus*, *G. caribaeus*, *G. carolinianus*, *G. carpenter*, *G. excentricus*, and *G. ribotype*. It is one of a series of marine polycyclic ether physiologically active toxins linked to ciguatera fish poisoning outbreaks [139]. However, it has also displayed therapeutic effects via increased muscular contraction, particularly in cardiac tissue and excessive fluid discharge by gastrointestinal cells [55,56]. Conversely, this biologically active toxin can be a useful tool for studying the biological function of a variety of human diseases and channelopathies, including cancer, chronic pain, epilepsy, and cardiac arrhythmias [140,141].

Maitotoxin (MTX) is a polyketide-derived polycyclic water-soluble molecule that has long been recognized as a possible aid in chemical and biological research [142,143]. This is the largest and most potent secondary metabolite ever isolated from the genus *Gambierdiscus* (*G. pacificus*, *G. australes*, and *G. toxicus*), and it comes in three different forms: MTX-1, MTX-2, and MTX-3 [144,145,146]. MTX is thought to be a powerful disruptor of Ca^2+^ homeostasis, with a wide range of pharmacological properties on a variety of cell lines [144]. It has the ability to initiate intracellular cascades of events such as membrane depolarization in excitable cells, insulin and neurotransmitter secretion, and phosphoinositide breakdown, which is imperative in cell lipids and cell signaling, programmed cell death, and fertilization, making it a useful tool for cell biology research, particularly when trying to understand Ca^2+^ dependent cellular developments [143,147,148,149]. In vivo, MTX seems to play a pivotal role in innate immune responses and inflammation in mice, making it a useful tool for studying specific aspects of the innate immune response and/or the physiology of inflammatory effector cells [144,150]. In *Xenopus laevis* oocytes, MTX was recently discovered to be a selective activator of an exact transient receptor potential (TRP) [151]. Maitotoxin promotes the synthesis and secretion of nerve growth factor by activating voltage-insensitive Ca^2+^ channels in C6-BU-1 glioma cells [151]. MTX could be useful in further research into these types of biological channels, as well as cancer, diabetes, and other stress-related human disorders [152].

Brevetoxin (BTX) is derived from the dinoflagellate *Karenia brevis* (formerly known as *Ptychodiscus brevis* or *Gymnodinium breve*) and has nine analogues that are categorized according to their backbone structure, such as type-A and type-B [153,154,155]. It reduces respiratory irritation symptoms such as cough, irritability of the nose, bronchoconstriction, congestion, and/or asthma attacks in people [156]. As a result, it changes the immune response in alveolar macrophage cells by boosting cytokines (TNF- and IL-2) implicated in immune cell activation, lowering phagocytosis activity and playing a crucial part in hypersensitivity inflammation in pulmonary tissue [57,157,158]. Furthermore, it has a dose-dependent effect on cell growth, causes cell death via apoptosis, and has genotoxic properties in Jurkat E6-1 cells and leukemic T-cell lines [57,58]. BTX-2 also exhibits neuro-activation qualities and can improve neuronal plasticity, which could be useful in pharmaceutical treatments for restoring brain function following a stroke or other traumatic brain damage [84]. A therapeutic invention based on BTX derivatives has also been developed to control disorders including cystic fibrosis and mucociliary dysfunction caused by amplification of mucus transport [159]. Paradoxical thermal dysthesia is a rare malfunction of the thermoregulatory system that happens in people who consume particular algal toxins. Mice are being studied to see how marine algae toxins such as maitotoxin and brevetoxin alter thermoregulatory processes. This type of research should lead to more effective treatments [160].

Zooxanthellatoxins (ZTs) A, B, and C are polyhydroxypolyenes with significant vasoconstrictive activity that have been identified from the cultivated dinoflagellate *Symbiodinium* sp. [161]. In addition, the amphoteric metabolites symbioimine and neosymbioimine are known to be produced by the same dinoflagellate genus. Symbioimine is an antiresorptive medication that can be used to prevent and treat osteoporosis in postmenopausal women [85]. Symbioimine may also be useful in the development of new nonsteroid anti-inflammatory medicines for the treatment of cyclooxygenase-2-related disorders [86].

Palytoxin (PLTX) is a complex polyether compound isolated from dinoflagellates such as *Ostreopsisfattorussoi*, *O. ovata*, *O. lenticularis*, *O. mascarenensis*, and *O. siamensis*, with notable biological activity, including a wide spectrum of pharmacological properties [162,163]. PLTX-like compounds formed by dinoflagellates are usually known as ostreocin. They modulates neurotransmitters (acetylcholine and/or norepinephrine) and activate pro-inflammatory signaling cascades such as the release of prostaglandin-E2 and histamine [164]. PLTX and ostreocin-D modulate cytoskeleton distortion and dynamics in intestinal and neuroblastoma cells and can significantly reduce cytotoxicity [60,61]. In addition, PLTX from *Palythoa clavata* polyps, comprising Symbiodinium dinoflagellate, displayed that a pharmaceutical formulation is appropriate for therapeutic use in the contradiction of lymphoblastic or myelogenous leukemia [59]. Discovery of novel properties of PLTX and PLTX like-compounds from marine dinoflagellates may lay the basis for a talented form of anti-cancer therapeutics.

*Gambierdiscus toxicus* dinoflagellate produces a toxin such as gambierol. Its chemical structure is similar to that of ciguatoxins and brevetoxins, and it has a high level of neurotoxicity [165,166]. Gambierol is also known as a CTX precursor [167]. Further biological research has been limited by its paucity of natural sources. Chemical synthesis has been attempted to address these challenges for in vitro and in vivo studies, and new immunotherapy medicines have been proposed [77,168,169]. Cao and colleagues discovered that gambierol causes bidirectional neurite development, which could be beneficial to patients with brain injury [87]. T-cell proliferation, immunological induction, and cytokine production are all induced by it, and it is thought to be a therapeutic target for T-cell-mediated autoimmune disorders [77,78]. Gambierol is an intriguing compound for its use as an immunosuppressant in diseases involving a malfunctioning immune system, such as multiple sclerosis, rheumatoid arthritis, and type 1 diabetes [77,78]. Gambierol and two of its analogues (tetra and heptacyclic forms) are potential compounds for reducing β-amyloid and/or tau hyperphosphorylation in Alzheimer’s disease both in vitro and in vivo [70]. Gambierol is an inhibitor of both PbTx-2-induced Ca^2+^ influx and cytotoxicity. Moreover, gambierol has been shown to be a potent antagonist of PbTx-2-induced Ca^2+^ and has been displayed as a functional antagonist of neurotoxin site 5 on neuronal VGSCs [170].

Azaspiracid (AZA) and its derivatives are phycotoxin polyethers generated by the *Azadinium* genus of dinoflagellates such as *A. dexteroporum*, *A. poporum*, and *A. spinosum* [171,172,173]. Azaspiracid-1 (AZA1), the first compound isolated and the one with the highest toxicity, is followed by AZA2 and AZA3 and has a significant biotechnological impact [174]. In vivo and in vitro toxicological investigations revealed cytotoxicity against a variety of human cell types as well as the capacity to modify cell shape and cytoskeleton structure, particularly in the E-cadherin system [136,175,176]. It was also discovered to be an active modulator of intracellular cAMP and calcium levels, as well as a potent activator of c-Jun-N-terminal kinase (JNK) and caspases, both of which are involved in stress-signaling pathways such as cytoskeleton regulation, cell damage, and apoptosis [177,178,179]. Furthermore, it lowers cell cholesterol levels, especially in T-lymphocyte cells [62,63].

Gymnocin-A (GYMA) is a rare toxin identified in *Gymnodinium mikimotoi*, a red tide dinoflagellate [180]. Although it is only mildly poisonous to fish, it is extremely toxic to P388 murine leukemia cells [64]. In the meantime, several additional variants of GYMA have been discovered, including Gymnocin-B, which has even developed cytotoxicity in several cell lines [64].

Karlotoxin (KmTx) is a linear polyketide toxin produced solely by the *Karlodinium* genus (*K. veneficum*). This biologically active toxin displayed a variety of actions, including haemolytic, cytotoxic, ichthyotoxic, and antifungal [65,80,81,82]. The biological activity of these chemicals is determined by the target cell’s sterol composition [65,181]. The ability of KmTx to cause the creation of pores in cholesterol-containing cell membranes suggests that it could be used to treat a variety of human diseases, including CHD (coronary heart disease). Furthermore, by inducing cell death through cholesterol depletion, KmTx could be developed as a new chemotherapeutic drug to control cancer in various solid tumor lines, such as prostate and breast cancer cells [65,66].

Spirolides (SPX) are biologically active toxins produced by *Karenia selliformes*, *Alexandrium ostenfeldii* and *A. peruvianum*, and there are currently 16 isoforms known [171,182,183,184,185]. SPX toxins have been shown to have a large deleterious effect [186]. Moreover, it displayed cytotoxic effects [186]. GYM (gymnodimine) and its two analogues Gymnodinoid dinoflagellates, notably *Karenia selliformis* (formerly known as *Gymnodinium selliforme*), make gymnodimine-A, GYM-B, and GYM-C [185,187]. GYM’s fourth analogue, 12-methylgymnodimine, was recently discovered as a novel analogue in *Alexandrium ostenfeldii* [188,189]. Spirolides have also been proven to have a neuroprotector role in Alzheimer’s [72]. These toxins can be used against different stress associated diseases.

**Figure 5:**
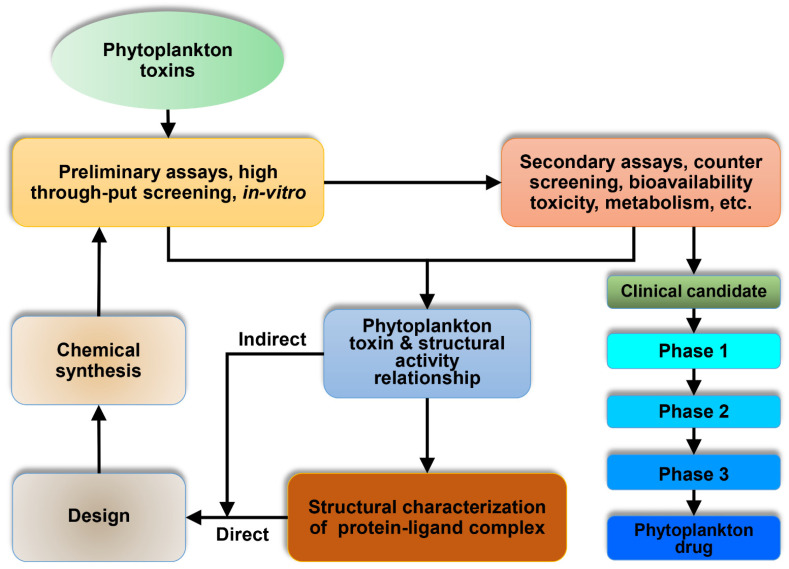
A proposed model for phytoplankton toxin as a future drug molecule. Toxins involve the identification of screening and optimization to increase the affinity through preliminary assays, high throughput screening, and in vitro screening. After successful screening, the phytoplankton toxin enters secondary assays, counter screening, bioavailability, toxicity, metabolism, etc. Then, screening of the phytoplankton toxin and its structural activity relationship can be performed through structural characterization of the protein–ligand complex. After conformation, it enters into modelling and designing of the toxin. Then, it enters into the chemical synthesis, which is more required for the clinical test. After the successful clinical phase is over, the phytoplankton toxin can be used as a drug.

Both SPXs and GYMs are found in *A. ostenfeldii* [190], are thought to have a pharmacophore component that activates L-type calcium channels in brain receptors and has a high affinity for neuronal and muscle nicotinic cholinergic receptors [187,191]. According to certain studies, the synergistic actions of GYM and OA can be employed therapeutically to boost anti-cancer effects by inducing tumor cell toxicity and acting as chemotherapeutic drugs. In the Neuro2a neuroblastoma cell line, GYM may also make cells more sensitive to apoptotic stimuli [67]. GYM may play a role in lowering amyloid levels and tau phosphorylation, which could help to treat degenerative illnesses [71]. Still, more research is required to explore dinoflagellate toxins in the field of pharmacology, even at a clinical level, to establish a new potent remedy against deadly diseases in humans such as cancer. Phytoplankton toxin can be used as a future drug molecule (Figure 5).

### 6.2. Bioactive Compounds from Dinoflagellates and Their Potential Biomedical Applications

Dinoflagellates are appealing sources of bioactive compounds for new drug progress by the pharmaceutical industry due to their extensive diversity and complexity in chemical structure [192]. Owing to their structural diversity of chemicals, the dinoflagellate bioactive compounds have been screened for several biomedical applications in different ROS associated diseases. Gambieric acid (GA) and its related gambieric acids A, B, C, and D were identified from the *Gambierdiscus toxicus* culture dinoflagellate [193]. They are effective antifungal drugs that have a high affinity for filamentous fungus but are ineffective against bacteria and yeasts. Furthermore, GA-A and GA-B have been shown to be 2000 times more effective than amphotericin B against the fungus *Aspergillus niger* [194]. GA, conversely, has no significant toxicity in cultivated mammalian cells or even in vivo [195].

*Alexandrium hiranoi*, *A. monilatum*, and *A. pseudogonyaulax* generate goniodomin A (GDA), which acts as an antifungal agent [196,197,198]. Pharmacological studies have shown that it has a significant impact on cytoskeleton remodeling [197]. By decreasing endothelial cell migration and basic fibroblast growth factor (bFGF)-induced tube formation via suppression of actin rearrangement, this drug limits angiogenesis (vessel regeneration). In vivo, GDA also inhibits angiogenesis [124]. GDA alters the actin state in astrocytoma cells, causing cell morphological changes by increasing filamentous actin [100]. GDA has been demonstrated to increase filamentous actin levels in clone 9 rat hepatocytes and to cause cytotoxicity in human neuroblastoma cells. A counterpart of GDA, goniodomin B, appears to have effects similar to GDA but is less powerful [199].

Amphidinolide (AMP) is generated by the dinoflagellate Amphidinium genus. Thus far, more than 40 AMPs have been found and show strong in vitro cytotoxicity against murine lymphoma L1210 and human epidermoid carcinoma KB cells [46,200]. Among all the AMPs, AMP-N has the strongest anti-tumor activity, with a preference for malignant cells’ mitochondria, while AMP-H appears to target the actin cytoskeleton [201]. This class of chemicals is likely to lead to new anticancer medicines, but their scarcity has prevented more comprehensive research [46,202]. A similar chemical, Caribenolide-I, was found to have a potent cytotoxic effect against a human colon carcinoma cell line and the murine tumor P388 [203].

Amphidinol (AM) is an antifungal and hemolytic compound generated by the Amphidinium genus, including *A. klebsii* and *A. carterae* [204]. Amphidinol 1 (AM1) was isolated from *A. klebsii* for the first time in 1991, and there have been around 23 AMs identified thus far, including seven analogues [205,206,207,208]. AMs are powerful cytotoxic compounds that can also promote proliferation and act as antifungal agents. AM3 had a stronger affinity for the ergosterol membrane, implying the production of a more stable complex, which could lead to the development of a new antifungal medication [209]. In addition, AM-5, derived from benthic Amphidinium species, promoted the proliferation of osteoblastic MC3T3-E1 cells and murine stromal ST-2 cells in the bone marrow [210]. Only at low doses did AM-4 promote highly intense proliferation in murine bone marrow stromal ST-2 cells, but not in MC3T3-E1 or NIH3T3 cells. It also improves the immune system’s ability by inducing TNF-α [211]. Iriomoteolide, another AM-related chemical discovered from Amphidinium benthic species, showed cytotoxic action against human cervical cancer HeLa cells [212].

Kobayashi et al. (1988) discovered a new form of biologically active ceramide, symbioramide, from the laboratory-cultured dinoflagellate *Symbiodinium* sp. [213], which showed antileukemic action in vitro against L-1210 murine leukemia cells [214]. Gambieric acids A–D, potent antifungal compounds derived from a culture of the marine dinoflagellate *Gambierdiscus toxicus* (GIII strain), have shown strong antifungal activity against filamentous fungus but are inert against yeasts [215]. Gambieric acids are up to 2000 times more effective than amphotericin B against some fungi. Gambieric acids are cytotoxic as well, although they do not have the same level of neurotoxicity as other big marine fused-polyether toxins such as ciguatoxins, brevetoxins, maitotoxins, and yessotoxins [194]. Additional research is needed to investigate the bioactive compounds found in dinoflagellates in order to discover a new cancer-fighting medicine and create a cancer-free environment. The need for a cancer-free and healthy environment is immense.

## 7. Conclusions and Future Prospects

Toxins derived from phytoplankton and their therapeutic interventions are briefly discussed. Phytoplankton have been proven to be a rich source of physiologically active toxins with intriguing biological properties that could be used in a variety of therapeutic and medicinal applications. Phytoplankton toxins are valuable in pharmacology because they contain a wide range of chemical structures as well as possess a wide range of biological properties. Despite their known value, the shortage of such biologically active toxins for more active research as well as preclinical testing, which may ultimately lead to commercial exploitation, continues to be a major problem. However, due to a paucity of pure toxins, several such toxins have not been well studied, and their pharmacological properties remain unknown. Future studies should be aimed at the synthesis of these toxins, such as an in-silico approach, the utilization of high-throughput technology, appropriate study design to implement desirable clinical trials, surface modification of the compounds, drug repurposing, and the formation of a noncomplex structure, as these will be highly relevant and sophisticated approaches for developing ideal and effective toxin molecules to be used for protection against diseases. More research is urgently needed to determine the precise mode of action of these unique physiologically and biologically active phytoplankton toxins and to develop potential pharmacophores against harmful diseases such as cancer and other diseases in humans.

## Figures and Tables

**Table 1:** Phytoplankton toxins and their different potential therapeutic applications.

Disease	Toxin	Application	Reference
Cancer	Okadaic acid (OA)	Breast, intestinal, blood, brain, lungs, hepatic, human leukemia and human endothelial cancer cell lines	[42,43,44,45]
	Amphidinolides and colopsinols	Murine lymphoma L1210 and human epidermoid carcinoma KB cells	[46]
	Caribenolide I	Human colon tumor cell line HCT 116 and HCT 116/VM 46	[47]
		in vivo against the mouse tumor P388	[48]
	Gymnocin-A	P388 murine leukemia cells	[49]
	Yessotoxins (YTXs)	Epithelial cancer cells	[50]
	YTX and its analogues	In BC3H1 myoblast cells, primary cortical neurons, and glioma cells	[51]
		Melanoma tumor cells	[52]
	Protoceratins I, II, III, and IV	Human colon cancer cell lines	[53]
	Pectenotoxin (PTX)	Lung, colon, and breast cancer cells	[54]
	Ciguatoxin (CTX)	Gastrointestinal cell lines	[55,56]
	Brevetoxin (BTX)	Jurkat E6-1 cell lines	[57,58]
	Palytoxin (PLTX)	Lymphoblastic or myelogenous leukemia cell lines	[59]
	Palytoxin (PLTX) and Ostreocin-D	Intestinal and neuroblastoma cell lines	[60,61]
	Azaspiracid (AZA)	T-lymphocyte cell lines	[62,63]
	Gymnocin-A (GYMA)	P388 murine leukemia cell lines	[64]
	Karlotoxin (KmTx)	Breast and prostate cancer cell lines	[65,66]
	Combination of GYM and OA	Several cancer cell lines	[67]
	GYM	Neuroblastoma cell line	[67]
Alzheimer	Okadaic acid (OA) YTX and its analogues Gambierol GYM Spirolides	Inhibits the level of t- and β-amyloid	[68] [69] [70] [71] [72]
Pain	Gonyautoxins (GTX)	-	[73]
	GTX2, GTX3 and TTX	-	[74]
Schizophrenia	Okadaic acid (OA)	-	[75]
Diabetes	Okadaic acid (OA) Gambierol	-	[76]
			[77,78]
AIDS	Okadaic acid (OA)	-	[76]
Fungal disease	Okadaic acid (OA) Karlotoxin (KmTx)	Suppress *Candida albicans* growth	[79]
			[65,80,81,82]
Allergy and Asthma	YTX and its analogues	-	[83]
Brain disorder	BTX-2	-	[84]
Osteoporosis	Symbioimine	Postmenopausal women	[85]
Inflammation	Symbioimine	Treatment of cyclooxygenase-2-related disorders	[86]
Brain injury, autoimmune disorders, multiple sclerosis, and rheumatoid arthritis	Gambierol	-	[87]
			[77,78]
			[77,78]
Coronary heart disease (CHD)	Karlotoxin (KmTx)	-	[65,66]
Pain,	Gonyautoxins (GTX)	-	[73]
Fungal, bacterial, and protozoal disease	Saxitoxin (STXs)	-	[88]

## Data Availability

Not applicable.

## Supplementary Materials

The following supporting information can be downloaded at: https://www.mdpi.com/article/10.3390/md20040271/s1, Supplementary Table S1: Toxins from dinoflagellate and their different potential therapeutic applications.

## Abbreviations

AM, Amphidinol; AMP, Amphidinolide; ATM, ataxia telangiectasia mutated; AZA, Azaspiracid; bFGF, basic fibroblast growth factor; BAX, Bcl-2-associated X protein; Bcl-2, B-cell lymphoma 2; Bcl-xl, B-cell lymphoma-extra-large; cAMP, cyclic adenosine monophosphate; BTX, Brevetoxin; CHD, coronary heart disease; CTX, Ciguatoxin; DA, domoic acid; DTX, dinophysistoxins; GA, Gambieric acid; GDA, goniodomin A; GTXs, gonyautoxins; GYM, gymnodimine; GYMA, Gymnocin-A; HIF-1, Hypoxia Inducible Factor-1; IL2, interleukin-2; JNK, c-Jun N-terminal kinase; KmTx, Karlotoxin; MTX, Maitotoxin; OA, Okadaic acid; OATPs, organic anion transporting polypeptides; PDE, potent phosphodiesterase; PP1 and PP2, protein phosphatase 1 and 2; PLTX, Palytoxin; PTXs, Pectenotoxins; ROS, reactive oxygen species; SPX, Spirolides; STX, saxitoxin; TNF, tumor necrosis factor, TTX, Tetrodotoxin; YTXs, Yessotoxins; ZTs, Zooxanthellatoxins.
