# Supplementary material for: Phytoplankton Toxins and Their Potential Therapeutic Applications: A Journey toward the Quest for Potent Pharmaceuticals
Source: Mar Drugs. 2022 Apr 18;20(4):271. doi: 10.3390/md20040271 (PMC9030253; doi:10.3390/md20040271)
Supplement: Supplementary file 1 [file marinedrugs-20-00271-s001.zip › marinedrugs-1657816-supplementary.pdf]

marine drugs

Supplementary Table S1: Toxins from dinoflagellate and their different potential therapeutic applications

Sl. No.	Toxin name	Source	Therapeutic action	References
1	Saxitoxin (STXs)	*Alexandrium minutum, Alexandrium tamarense, Alexandrium catenella, Gymnodinium. catenatum,* and *Pyrodinium bahamense,*	Displayed *in vitro* antifungal, antibacterial, antialgal and antiprotozoal activity	[88]
2	Gonyautoxins (GTX)	*Amphydinium*	Used as a pain reliever	[73]
	GTX2 and GTX3	*Amphydinium*	Chronic tension-type headaches	[74]
3	Tetrodotoxin (TTX)	*Alexandrium tamarense*	Reduce narcotic cravings in laboratory animals and is being employed in drug addiction. Treatment of heroin addiction withdrawal	[122]
4	Okadaic acid (OA)	*Porocentrum lima, Porocentrum cuncavum, Porocentrum belezeanum, Porocentrum mascul*	Cytotoxic effects in numerous cell types, including intestinal cells, blood cells, brain cells, lung cells, and hepatic cells,	[43]
4	Okadaic acid (OA)	*Porocentrum lima, Porocentrum cuncavum, Porocentrum*	Act as anti-tumor agents on breast cancer cells as it inhibits protein phosphatase 2A	[42]
5	Okadaic acid (OA)	*Porocentrum lima, Porocentrum cuncavum, Porocentrum*	Act as typical antipsychotic medications in the treatment of cognitive impairment and neuropathological alterations in schizophrenia and other neurodegenerative illnesses because of its tumor-promoting and cytotoxic actions	[75]
6	Okadaic acid (OA)	*Porocentrum lima, Porocentrum cuncavum, Porocentrum*	Alzheimer's disease and other neurodegenerative illnesses linked to memory loss	[68]
7	Okadaic acid (OA)	*Porocentrum lima, Porocentrum cuncavum, Porocentrum*	Diabetes, AIDS, and cancer to reveal numerous pathways related to these diseases	[76]
8	Okadaic acid (OA)	*Porocentrum lima, Porocentrum cuncavum, Porocentrum*	Immunoregulatory potential, since it causes T-cell receptor expression to be	[128]

			downregulated, affecting T-cell responsiveness and, as a result, immunological response	
9	Okadaic acid (OA)	*Porocentrum lima, Porocentrum cuncavum, Porocentrum*	Triggers an inflammatory response in HL-60 human cells by significantly increasing interleukin 8 (IL-8) levels	[44]
10	Okadaic acid (OA)	*Porocentrum lima, Porocentrum cuncavum, Porocentrum*	Increase the activity of hypoxia-inducible factor-1 (HIF-1) a protein closely linked to vascular endothelial growth factor in human endothelial cells	[45]
11	Okadaic acid (OA)	*Porocentrum lima, Porocentrum cuncavum, Porocentrum*	Fungicidal properties, specifically the capacity to suppress Candida albicans growth	[79]
12	Amphidinolides and colopsinols	*Amphidinium*	Exhibit powerful anticancer effects via inhibition of cancer cell lines	[129]
13	Amphidinolides and colopsinols	*Amphidinium*	Showed high cytotoxicity against murine lymphoma L1210 and human epidermoid carcinoma KB cells	[46]
14	Caribenolide I	*Amphidinium*	Showed high cytotoxicity in human colon tumor cell line HCT 116 and its drugresistant ariation HCT 116/VM 46	[47]
15	Caribenolide I	*Amphidinium*	Showed efficacious *in vivo* against the mouse tumor P388	[48]
16	Gonyautoxins	*Amphidinium*	It has been employed in clinical practice for anal sphincter infiltrations	[73]
17	Goniodomin-A	*Goniodoma pseudogoniaulax*	Prevent angiogenesis by decreasing endothelial cell migration and tube formation caused by basic fibroblast growth factor (bFGF)	[124]
18	Gymnocin-A	*Gymnodinium mikimotoi*	Displayed cytotoxic to P388 murine leukemia cells	[49]
19	Yessotoxins (YTXs)	*Protoceratium reticulatum, Lingulodinium polyedra,* and *Gonyaulax spinifera*	Impair the E-cadherin–catenin system selectively in epithelial cells, thus jeopardizing Ecadherin's tumor-suppressive effects	[50]

20	Yessotoxins (YTXs)	*Protoceratium cf. reticulatum*	Displayed significant cytotoxic effects	[120]
21	Protoceratins I, II, III, and IV	*Protoceratium cf. reticulatum*	Displayed cytotoxicity selectively against human cancer cell lines with mean IC_{50} values of less than 0.0005 M	[53]
22	YTX and its analogues	*Protoceratium reticulatum, Lingulodinium polyedra,* and *Gonyaulax spinifera*	Diplayed multiple biological apoptotic pathways	[131]
23	YTX and its analogues	*Protoceratium reticulatum, Lingulodinium polyedra,* and *Gonyaulax spinifera*	In BC3H1 myoblast cells, primary cortical neurons, and glioma cells, it also causes non-apoptotic cell death	[51]
24	YTX and its analogues	*Protoceratium reticulatum, Lingulodinium polyedra,* and *Gonyaulax spinifera*	Modulates caspase protein activation via permeability transition through mitochondria and alteration of cytoskeleton (viz. selective disruption of F-actin microfilaments)	[133,134]
25	YTX and its analogues	*Protoceratium reticulatum, Lingulodinium polyedra,* and *Gonyaulax spinifera*	Inhibits the growth of melanoma tumor cells in mice cells *in vivo*, with minimal damage	[52]
26	YTX and its analogues	*Protoceratium reticulatum, Lingulodinium polyedra,* and *Gonyaulax spinifera*	Impair immunological function by reducing phagocytic activity in the J774 cell line and increasing cytokine expression in J774 phagocyte mammalian cells	[133]
27	YTX and its analogues	*Protoceratium reticulatum, Lingulodinium polyedra,* and *Gonyaulax spinifera*	Control the immunological impact on T-lymphocyte EL-4 cells via reversible T-cell receptor complex down-regulation	[128]
28	YTX and its analogues	*Protoceratium reticulatum, Lingulodinium polyedra,* and *Gonyaulax spinifera*	Used to treat Alzheimer's disease by lowering the levels of t- and β-amyloid, two insoluble formations found in the brain that are responsible for the illness's onset	[69]
29	YTX and its analogues	*Protoceratium reticulatum, Lingulodinium polyedra,* and *Gonyaulax spinifera*	Treatment/prevention of lipid and glucose metabolism-related disorders in glioma cells, as well as pancreatic and liver transcriptional abnormalities	[135]

30	YTX and its analogues	*Protoceratium reticulatum, Lingulodinium polyedra,* and *Gonyaulax spinifera*	Minor role as anti-allergenic and -asthmatic drug	[83]
31	Pectenotoxin (PTX)	*D. fortii, D. acuta, D. tripus, D. acuminate, D. caudate, D. rotundata, and D. norvegica*	Strong cytotoxic against various human cancer cell lines such as lung, colon, and breast cancer cells	[54]
32	Pectenotoxin (PTX)	*D. fortii, D. acuta, D. tripus, D. acuminate, D. caudate, D. rotundata, and D. norvegica*	Act as potential chemotherapeutic treatment for p53-deficient malignancies	[138]
33	Ciguatoxin (CTX)	*Gambierdiscus toxicus*	Increased muscular contraction, particularly in cardiac tissue, and excessive fluid secretion by gastrointestinal cells	[55,56]
34	Maitotoxin (MTX)	*Gambierdiscus toxicus, G. australes, and Gambierdiscus pacificus*	Displayed innate immune responses and inflammation	[144,150]
35	Maitotoxin (MTX)	*Gambierdiscus toxicus, G. australes, and Gambierdiscus pacificus*	Promotes the synthesis and secretion of nerve growth factor by activating voltage-insensitive Ca2+ channels in C6-BU-1 glioma cells	[151]
36	Brevetoxin (BTX)	*Karenia brevis (formerly known as Gymnodinium breve or Ptychodiscus brevis)*	Reduces respiratory irritation symptoms such as cough, irritability of the nose, congestion, bronchoconstriction, and/or asthma attacks in people	[156]
37	Brevetoxin (BTX)	*Karenia brevis (formerly known as Gymnodinium breve or Ptychodiscus brevis)*	Alters immunological response in alveolar macrophage cells by increasing cytokines (such as TNF- and IL-2) involved in immune cell activation, which reduces phagocytosis activity and plays a key role in allergic inflammation in pulmonary tissue	[57,157,158]
38	Brevetoxin (BTX)	*Karenia brevis (formerly known as Gymnodinium breve or Ptychodiscus brevis)*	Displayed a dose-dependent effect on cell growth, causes cell death via an apoptotic mechanism, and has genotoxic propertiesn	[57,58]
39	BTX-2	*Karenia brevis (formerly known as Gymnodinium breve or Ptychodiscus brevis)*	It can improve neuronal plasticity and treatments for restoring brain function	[84]

			following a stroke or other traumatic brain damage	
40	Zooxanthellatoxins (ZTs) A, B, and C	*Symbiodinium* sp.	Vasoconstrictive activity	[161]
41	Symbioimine	*Symbiodinium* sp.	Displayed an antiresorptive medication that could be used to prevent and treat osteoporosis in postmenopausal women	[85]
42	Symbioimine	*Symbiodinium* sp.	May also be useful in the development of new nonsteroid anti-inflammatory medicines for the treatment of cyclooxygenase-2-related disorders	[86]
43	Palytoxin (PLTX)	*Ostreopsis siamensis, Ostreopsis mascarenensis, Ostreopsis lenticularis, Ostreopsis ovata, Ostreopsis fattorussoi*	Remarkable biological activity including a wide spectrum of pharmacological effects	[162,163]
44	Ostreocin-D	*Ostreopsis siamensis, Ostreopsis mascarenensis, Ostreopsis lenticularis, Ostreopsis ovata, Ostreopsis fattorussoi*	Modulates neurotransmitters (norepinephrine and/or acetylcholine), and activates pro-inflammatory signaling cascades such as release of histamine and prostaglandin-E2	[164]
45	PLTX and Ostreocin-D	*Ostreopsis siamensis, Ostreopsis mascarenensis, Ostreopsis lenticularis, Ostreopsis ovata, Ostreopsis fattorussoi*	Modulates cytoskeleton distortion and dynamics in intestinal and neuroblastoma cells and also can significantly reduce cytotoxicity	[60,61]
46	PLTX	*Palythoa clavata polyps*	Suitable for therapeutic use against lymphoblastic or myelogenous leukemia	[59]
47	Gambierol	*Gambierdiscus toxicus*	Beneficial to patients of brain injury	[87]
48	Gambierol	*Gambierdiscus toxicus*	T-cell-mediated autoimmune disorders	[77,78]
49	Gambierol	*Gambierdiscus toxicus*	Used against multiple sclerosis, type 1 diabetes, and rheumatoid arthritis	[77,78]
50	Gambierol	*Gambierdiscus toxicus*	Reducing β-amyloid and/or tau hyperphosphorylation in Alzheimer's disease both *in vitro* and *in vivo*	[70]

51	Azaspiracid (AZA)	*Azadinium spinosum, Azadinium poporum,* and *Azadinium dexteroporum*	Cytotoxicity against a variety of human cell types, as well as the capacity to modify cell shape and cytoskeleton structure, particularly in the E-cadherin system	[136,175,176]
52	Azaspiracid (AZA)	*Azadinium spinosum, Azadinium poporum,* and *Azadinium dexteroporum*	Effective modulator of intracellular cAMP and calcium levels, as well as a potent activator of c-Jun-N-terminal kinase (JNK) and caspases, both of which are involved in stress-signaling pathways such as cell damage, apoptosis, and cytoskeleton regulation	[177-179]
53	Azaspiracid (AZA)	*Azadinium spinosum, Azadinium poporum,* and *Azadinium dexteroporum*	Lowers cell cholesterol levels, especially in T-lymphocyte cells	[62,63]
54	Gymnocin-A (GYMA)	*Gymnodinium mikimotoi*	Extremely toxic to P388 murine leukemia cells.	[64]
55	Karlotoxin (KmTx)	Karlodinium veneficum	Displayed variety of actions, including haemolytic, cytotoxic, ichthyotoxic, and antifungal	[65,80-82]
56	Karlotoxin (KmTx)	*Karlodinium veneficum*	Used to treat a variety of human diseases, including coronary heart disease (CHD)	[65,66]
57	Karlotoxin (KmTx)	*Karlodinium veneficum*	Used as new chemotherapeutic drug to control cancer in various solid tumor lines, such as breast and prostate cancer cells, by inducing cell death through cholesterol depletion	[65,66]
58	Spirolide (SPX)	*Alexandrium ostenfeldii/peruvianum* and *Karenia selliformes*	Cytotoxic effects	[186]
59	Combination of GYM and OA		Employed therapeutically to boost anticancer effects by inducing tumor cell toxicity and acting as chemotherapeutic drugs	[67]
60	GYM		More sensitive to apoptotic stimuli. Neuro2a neuroblastoma cell line	[67]

61	GYM	Lowering -amyloid levels and tau phosphorylation, which could help with the treatment of degenerative illnesses	[71]
